# Electrospinning on 3D Printed Polymers for Mechanically Stabilized Filter Composites

**DOI:** 10.3390/polym11122034

**Published:** 2019-12-08

**Authors:** Tomasz Kozior, Al Mamun, Marah Trabelsi, Martin Wortmann, Sabantina Lilia, Andrea Ehrmann

**Affiliations:** 1 Faculty of Mechatronics and Mechanical Engineering, Kielce University of Technology, 25-314 Kielce, Poland; 2 Faculty of Engineering and Mathematics, Bielefeld University of Applied Sciences, 33619 Bielefeld, Germany; al.mamun@fh-bielefeld.de (A.M.); marah.trabelsi@enis.tn (M.T.); martin.wortmann@fh-bielefeld.de (M.W.); lilia.sabantina@fh-bielefeld.de (S.L.); andrea.ehrmann@fh-bielefeld.de (A.E.); 3 Ecole Doctorale Science et Technologies, Ecole Nationale d’Ingénieurs de Sfax, Sfax 3038, Tunisia

**Keywords:** electrospinning, 3D printing, FDM printing, nanofiber mat, adhesion, water filter

## Abstract

Electrospinning is a frequently used method to prepare air and water filters. Electrospun nanofiber mats can have very small pores, allowing for filtering of even the smallest particles or molecules. In addition, their high surface-to-volume ratio allows for the integration of materials which may additionally treat the filtered material through photo-degradation, possess antimicrobial properties, etc., thus enhancing their applicability. However, the fine nanofiber mats are prone to mechanical damage. Possible solutions include reinforcement by embedding them in composites or gluing them onto layers that are more mechanically stable. In a previous study, we showed that it is generally possible to stabilize electrospun nanofiber mats by 3D printing rigid polymer layers onto them. Since this procedure is not technically easy and needs some experience to avoid delamination as well as damaging the nanofiber mat by the hot nozzle, here we report on the reversed technique (i.e., first 3D printing a rigid scaffold and subsequently electrospinning the nanofiber mat on top of it). We show that, although the adhesion between both materials is insufficient in the case of a common rigid printing polymer, nanofiber mats show strong adhesion to 3D printed scaffolds from thermoplastic polyurethane (TPU). This paves the way to a second approach of combining 3D printing and electrospinning in order to prepare mechanically stable filters with a nanofibrous surface.

## 1. Introduction

Filters are used to separate water from oil, biological and inorganic material from water or gas fluids, etc. Several technologies are used, depending on the exact application, such as granular filtration with quartz sand [1], superhydrophobic meshes for water/oil separation [2], reactive superhydrophobic paper for water filtration [3], molecular sieves from graphene oxide for nanofiltration [4] or reverse osmosis for water cleaning in power plants [5].

Another large research area of filter research is based on electrospun nanofiber mats [6,7,8,9,10,11]. Electrospinning uses a strong electric field, either between a needle and a substrate or between two electrodes in the form of wires, cylinders, or other shapes, to prepare nanofibers from polymers or polymer blends [12,13,14]. Although such nanofiber mats offer a large specific surface area and are therefore ideally suited for filters and other applications necessitating a large contact area with the environment, their mechanical stability is usually low. Due to areal weights one or two orders of magnitude below the values for common copy paper, use in harsh environments such as in water filtration is a difficult challenge [15,16]. To solve this problem, Qin and Wang suggested crosslinking and inclusion in textile composites [17], and Fu et al. found chemical crosslinking sufficient for use in lithium-ion batteries [18]. Roche and Yalcinkaja prepared functional filter structures with integrated nanofiber mats [19], whereas other groups suggested the coating [20], heat pressing [20,21], or ultrasonic welding of several nanofiber mats [22].

Recently, we suggested another approach. By 3D printing with the fused deposition modeling (FDM) technology on electrospun nanofiber mats, we prepared composites with a nanofiber layer on top of a rigid polymer layer that showed significantly increased abrasion resistance compared to the pure nanofiber mat, without significant modification of the measured contact angles [23].

Although 3D printing on textile surfaces is a frequently investigated research topic [24,25,26,27,28,29,30,31,32], 3D printing on nanofiber mats has proved to be highly challenging. Therefore, we describe the reverse approach here.

Generally, research on FDM printing mostly concentrates on surface roughness, dimensional accuracy, and mechanical properties [33,34,35,36,37]. The adhesion of a material placed on the FDM printed surface is scarcely investigated, especially with electrospinning as the second step. Yeo and Kim reported on electrospinning alginate/poly(ethylene oxide) nanofibers on a poly(ε-caprolactone) strut prepared by dispensing 3D printing [38]. Rajzer et al. used electrospinning with a gelatin solution on poly(l-lactic acid) 3D printed scaffolds [39], while Naghieh et al. performed electrospinning of gelatin on a poly(lactic acid) scaffold [40]. Mendoza-Buenrostro et al. even prepared a combined apparatus, allowing for alternate 3D printing and electrospinning, to create substrates for tissue engineering [41]. However, these applications all concentrate on tissue engineering. The composites prepared in the aforementioned studies are not necessarily suited for water filtration. Here we report on electrospinning on objects 3D printed from a rigid and a flexible filament, the resulting adhesion, and finally the resulting abrasion properties of the composites.

## 2. Materials and Methods

3D-printed objects were prepared from poly(lactic acid) (PLA) (Filamentworld, Neu-Ulm, Germany) and the thermoplastic polyurethane (TPU) Filaflex (Filamentworld). Printing was performed by the FDM printer Orcabot XXL (Prodim, Helmond, The Netherlands), using a nozzle diameter of 0.4 mm, a layer thickness of 0.2 mm, a nozzle temperature of 190 °C (PLA) or 240 °C (Filaflex), and a printing bed temperature of 60 °C. All objects had a thickness of 0.4 mm (i.e., two layers). Both layers were printed with three perimeter lines and rectilinear filling under an angle of ±45°, applying a flow rate of 100%.

The 3D-printed objects were glued onto the polypropylene substrate, which is typically used for electrospinning in the needleless electrospinning machine “Nanospider Lab” (Elmarco Ltd., Liberec, Czech Republic). The following spinning parameters were used: voltage 80 kV, nozzle diameter 0.8 mm, carriage speed 100 mm/s, bottom electrode/substrate distance 240 mm, ground electrode/substrate distance 50 mm, temperature in the chamber 22–23 °C, and relative humidity in the chamber 32%. Spinning was carried out for 8 min per sample, resulting in a relatively fine nanofiber mat which breaks easily when removed from the substrate (areal weight 1.7–2.1 g/m²). For some of the experiments, conductive nanofiber mats were glued onto the polypropylene substrate as a pre-test for electrospinning on a conductively coated 3D printed object from PLA. In both cases, the conductive coating of PLA or the nanofiber mat was achieved by applying PEDOT:PSS (purchased from Sigma-Aldrich Chemie GmbH, Munich, Germany) on the surface and letting it air dry.

The electrospinning solution consisted of 16% polyacrylonitrile (PAN) dissolved in dimethyl sulfoxide (DMSO, min. 99.9 %, S3 Chemicals, Bad Oeynhausen, Germany). PAN can be used as a filter material [21], is spinnable from the low-toxicity solvent DMSO [42], and shows well-defined dependencies of nanofiber mat thickness, fiber diameters, and other morphological properties on the spinning and solution parameters [43].

The optical evaluation of the composites was performed with a digital microscope VHX-600D (Keyence, Neu-Isenburg, Germany) and a confocal laser scanning microscope (CLSM) VK-8710 (Keyence). To perform contact angle measurement, drops of distilled water (volume 15 µL) were placed on the samples under investigation, then microscopic images were taken with the digital microscope and used to fit the contact angles between the drop contour and baseline.

A Martindale abrasion tester (self-built) was used to investigate the abrasion of the nanofiber mats on the 3D printed objects. In accordance with ISO 12947, the damage on the surface was investigated with a digital microscope.

Tensile tests were performed using samples of width 50 mm, length 140 mm (free clamping length 100 mm), and thickness 0.4 mm (i.e., two layers identical to the electrospinning samples). The samples were elongated in a Sauter TVM-N universal testing machine (Kern & Sohn GmbH, Balingen-Frommern, Germany). Maximum tensile tests were performed with a maximum elongation of 108% (the maximum value possible in the tensile tester). Repeated tensile tests were performed with an elongation of up to 25% for 10 test cycles. In both cases, the testing speed was 80 mm/min.

Differential scanning calorimetry (DSC) was performed on a DSC 3 device by Mettler-Toledo (Gießen, Germany) with a heating rate of 10 K/min under nitrogen atmosphere.

The chemical composition was investigated by a Fourier transform infrared (FTIR) spectrometer Excalibur 3100 (Varian Inc., Palo Alto, CA, USA).

## 3. Results and Discussion

**Figure 1:**
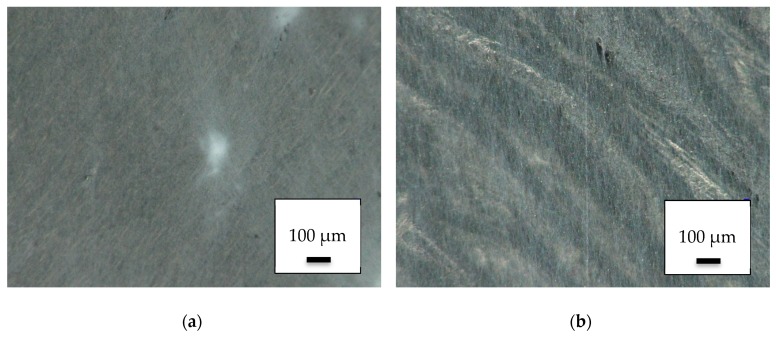
Nanofiber mats on 3D-printed poly(lactic acid) (PLA) objects: (**a**) dense area near the middle of the electrospinning area; (**b**) less dense area near the outer border of the electrospinning area.

Although 3D printing on electrospun nanofiber mats is quite challenging [23], the reversed process is not technologically complex. Electrospinning on a 3D printed PLA object resulted in the surface depicted in Figure 1, showing one of the denser (Figure 1a) and one of the less dense regions in which the 3D-printed surface is visible below the nanofibers (Figure 1b).

**Figure 2:**
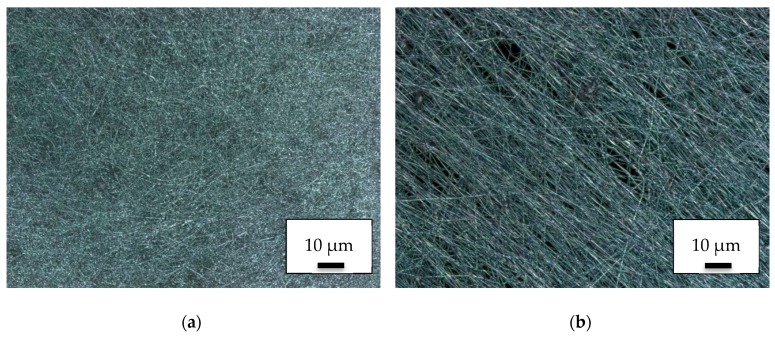
CLSM images of nanofiber mats on 3D-printed PLA objects: (**a**) dense area near the middle of the electrospinning area; (**b**) less dense area near the outer border of the electrospinning area.

Investigating the same areas by CLSM, the nanofiber mat showed a denser and isotropic structure near the center (Figure 2a). Along the outer borders the nanofibers showed a typical orientation due to the electric field which was laterally anisotropic there (Figure 2b). In both cases, the spinning process did not differ from the results of direct electrospinning onto common polypropylene substrates.

**Figure 3:**
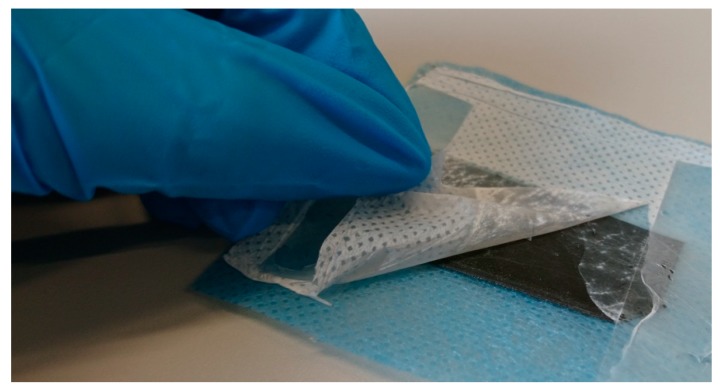
Pulling a nanofiber mat away from the 3D-printed PLA substrate below.

The most important factor determining the applicability of such composites as filters, however, is the adhesion between both materials. As Figure 3 reveals, the adhesion of the nanofiber mats onto the PLA printed object is very low. Detaching the glue directly resulted in separation of the nanofiber mat from the 3D printed objects, regardless of the position of the latter during electrospinning and the side (top or bottom) on which electrospinning was performed.

Next, the influence of a conductive substrate prepared by coating 3D printed PLA objects with PEDOT:PSS on the electrospinning process was investigated. Integrating conductive areas modifies the electric field applied on the nanofibers during their flight to the substrate and may possibly result in a difference in adhesion.

**Figure 4:**
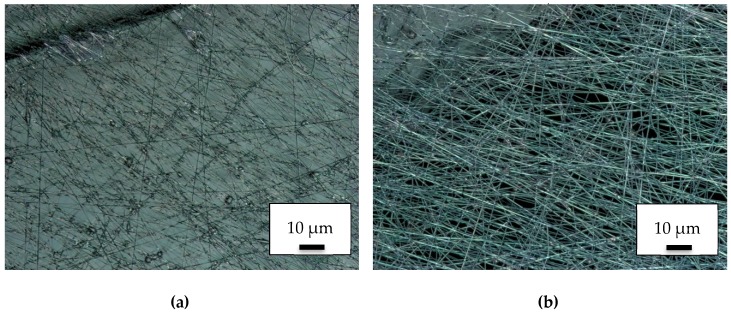
CLSM images of nanofiber mats on conductive 3D printed PLA objects: (**a**) on top of the conductive PLA object; (**b**) directly beside the conductive object.

However, the influence of this process, was opposite to that intended. As the CLSM images in Figure 4a (taken on the conductively coated PLA object) and 4b (taken directly beside the corner of the conductive part) show, the fiber formation was significantly reduced on the conductive substrates. At the same time, the adhesion did not change. This approach was thus rejected.

Since previous experiments with the 3D printing of different materials onto each other have revealed strongly different adhesion for printing with a flexible material on a rigid one and vice versa, the next series of experiments was performed on objects 3D printed from flexible TPU filament.

**Figure 5:**
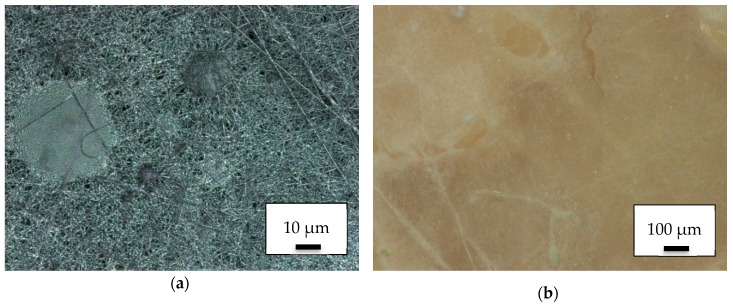
PAN electrospun onto a thermoplastic polyurethane (TPU) object: (**a**) CLSM image; (**b**) microscopic image.

Figure 5 depicts such a sample surface. Apparently, on both scales shown here, the electrospun nanofiber mat was less even than for electrospinning on the rigid PLA substrate or on the common nonwoven polypropylene. This finding can most probably be attributed to the less even surface of the object 3D printed with the elastic material.

More interesting, however, is the adhesion between the electrospun nanofiber mat and the 3D printed surface below. First tests clearly show that the nanofiber mat cannot be pulled apart from the TPU substrate. This difference can be attributed to the different interaction between the 3D printed substrates and the residues of the solvent DMSO, which can still be expected to be slightly available in the PAN nanofiber mat after being positioned on the substrate. Although PLA does not react with DMSO, the TPU Filaflex is dissolved in DMSO. In this way, a connection between the nanofiber mat and the TPU substrate can be formed, which is impossible on PLA objects.

This suggests performing Martindale abrasion tests on the pure nanofiber mats and on the nanofiber mats as composites on top of the TPU objects.

Due to the short spinning duration of only 8 min per sample, the pure nanofiber mats broke after one Martindale abrasion cycle, independent of whether they are in a dry or wet state. This was even less than in the previous examination on nanofiber mats that were approx. twice as thick [23] which could withstand 5–10 Martindale cycles in the dry state, but were also destroyed after one Martindale cycle in the wet state. 

**Figure 6:**
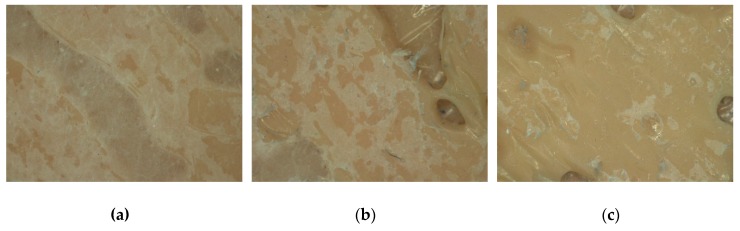
Microscopic images of composite surface after Martindale abrasion tests taken (**a**) after 1 Martindale cycle; (**b**) after 20 cycles in dry state; (**c**) after 100 cycles in dry state.

Figure 6 shows microscopic images after 1, 20, and 100 Martindale cycles, respectively. While certain irregularities in the nanofiber mat density were already visible after the first Martindale cycle (Figure 6a), these irregularities became stronger after 20 cycles (Figure 6b). The test was stopped after 100 Martindale cycles when in most areas nanofibers were no longer visible (Figure 6c).

Although the composite formation could therefore significantly increase the abrasion resistance of nanofiber mats, more detailed investigations are necessary in the future to further optimize this effect. First, it is necessary to improve the 3D printing process to gain a more even surface or a well-defined roughness which may even further increase the protective effect of the composite formation. On the other hand, for the planned application in water filters, fluid dynamic calculations are necessary alongside the pure material science part, to optimize the filtering properties of such composites in aqueous environments.

**Figure 7:**
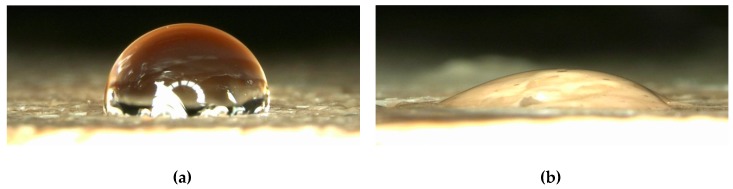
Contact angles, investigated by dropping 15 µL of distilled water on (**a**) an FDM-printed TPU layer and(**b**) a PAN/TPU composite.

Since wettability is a defining property for filter applications, contact angle measurements were performed. Figure 7 shows contact angle measurements on a pure TPU layer (Figure 7a) and on the PAN/TPU composite surface (Figure 7b), respectively. Although the original PAN nanofiber mat exhibited a contact angle of (31 ± 3)° [23], a hydrophobic contact angle of (99 ± 2)° was measured on the pure TPU layer. With (36 ± 4)°, the composite showed a slightly increased contact angle as compared to the pure nanofiber mat. Opposite to the previously investigated PAN/PLA composite surfaces, here no directional dependence of the water spreading in the composite surface could be recognized, making the softer PAN/TPU composite possibly better suited for water filtration, since the orientation with respect to the direction of water flow likely causes less variance.

**Figure 8:**
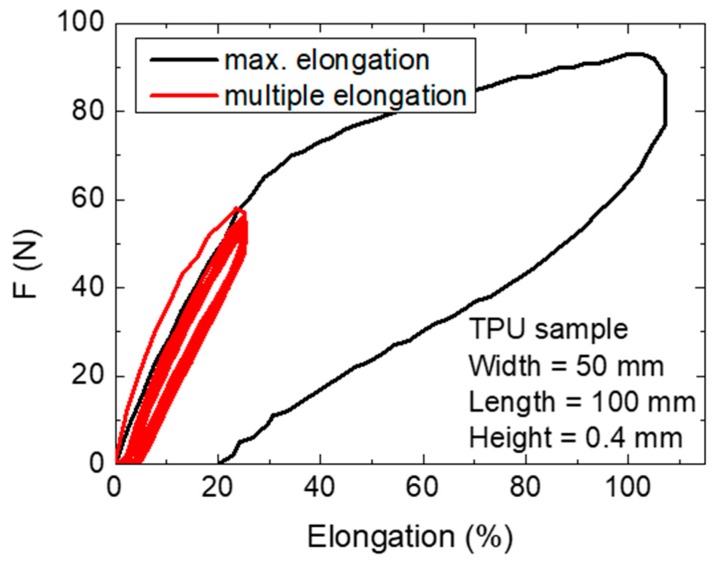
Tensile tests with the maximum elongation available in the tensile tester and with multiple elongation cycles.

To also examine the mechanical properties of the TPU substrate, samples were subjected to elongation tests, the results of which are given in Figure 8.

As expected, the highly elastic TPU samples could be stretched by more than 100% without breaking. The values for repeated measurements of only 25% elongation also showed a hysteresis; the curves became nearly identical after few repetitions. After 1 h at rest, the material had relaxed and again started with the first curve offset from those following.

This experiment is not possible with pure PAN nanofiber mats, which usually break at forces below 1 N, the resolution limit of our tensile tester. Accordingly, the nanofiber mat had no influence on the mechanical properties of the TPU substrate, yet the latter can significantly stabilize the first.

It is also necessary to mention that the nanofiber mat can only be stretched by a few percent before breakage or severe, undesired deformation. This means that, depending on the forces imposed on the composite suggested here, the composite itself must also be backed with a non-stretchable substrate to avoid breaking of the nanofiber mat. However, this is not critical for the suggested use in fluid filters.

**Figure 9:**
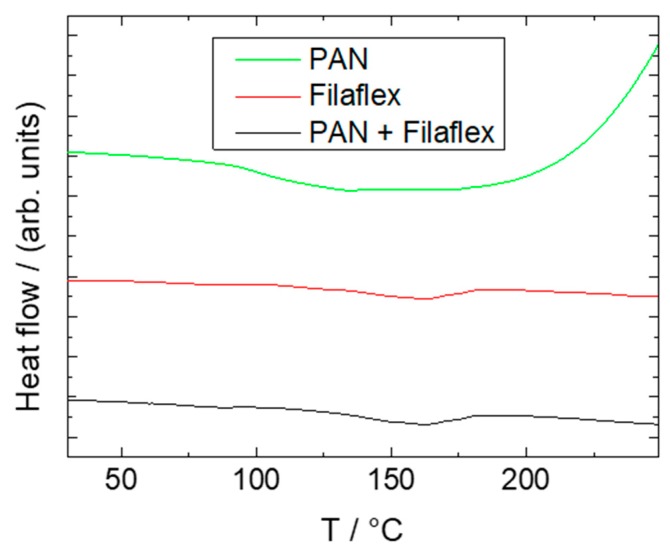
DSC measurements of a pure PAN nanofiber mat, pure Filaflex TPU, and a PAN/Filaflex composite. The lines are vertically offset for clarity.

Figure 9 shows DSC measurements from 25 to 250 °C at 10 K/min of pure TPU, TPU covered with PAN nanofibers, and pure PAN nanofibers. As usual for FDM materials, TPU exhibited a rather high onset glass transition temperature of around 132 °C, ensuring quick solidification on previous layers. As can be seen, there was no significant difference between pure and covered TPU, which is not surprising given that that the ultra-thin nanofiber mat covering the surface contributed almost nothing to the overall sample mass. Pure PAN nanofibers showed an onset glass transition temperature of 93 °C, which is in good agreement with known values. At around 200 °C the PAN fibers appeared to show the beginning of an exothermic cyclization reaction, as commonly used for pretreatment for subsequent carbonization [44]. A subsequent thermal treatment of as-spun nanofibers between both glass transition temperatures might be suitable to enhance adhesion between the fibers and the printing material without compromising the overall shape accuracy.

**Figure 10:**
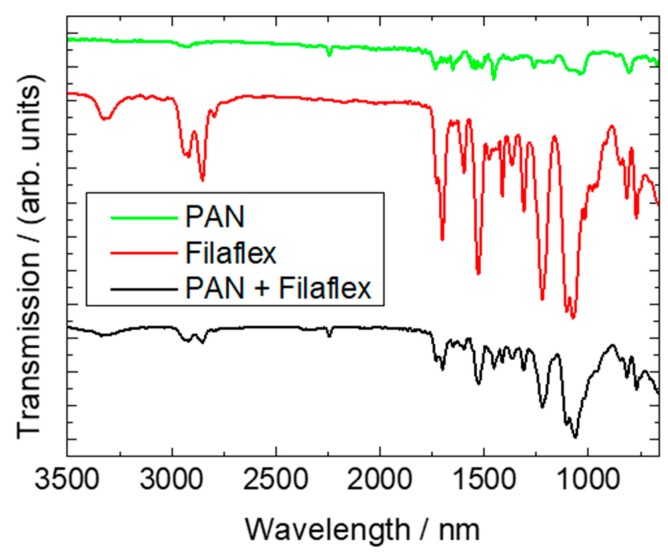
FTIR measurements of a pure PAN nanofiber mat, pure Filaflex, and a PAN/Filaflex composite. The lines are vertically offset for clarity.

Finally, FTIR measurements were performed. These are more surface-sensitive than DSC and thus can be expected to show a difference between the pure TPU Filaflex and the TPU/PAN composite. The results are depicted in Figure 10.

The spectrum of PAN shows the usual lines [45], such as the characteristic stretching vibration of the C≡N nitrile functional group at 2240 cm^−1^ and the carbonyl (C=O) stretching peak at 1732 cm^−1^. The peaks around 1230–1250 cm^−1^ and between 1050–1090 cm^−1^ can be identified as ester (C–O and C–O–C) vibrations of the co-monomers like itaconic acid or methyl acrylate, typical materials used in industrial PAN production [45]. Near 2938 cm^−1^, 1452 cm^−1^, and 1380 cm^−1^, bending and stretching vibrations of CH_2_ are visible [45].

Different peaks occurred for the Filaflex. The peak around 3328 cm^−1^ can be attributed to N–H, and the double peak between 2800 and 3000 cm^−1^ stems from CH_2_ [46]. Next, the peak around 1701 cm^−1^ is correlated with C=O stretching, followed by the peak near 1595 cm^−1^ according to the typical phenyl ring of TPU elastomers [46]. Near 1540 cm^−1^, C–N stretching and N–H deformation become visible, and the double-peak around 1162 cm^−1^ and 1076 cm^−1^ can be attributed to C–O–C stretching [46].

Measuring the composite surface revealed a combination of both materials. The characteristic peaks of both materials could be found again in reduced form, revealing that no unexpected chemical modifications occur during electrospinning, which would be visible by additional peaks in the FTIR graph of the composite.

## 4. Conclusions

In this study, we have shown that, although electrospinning PAN nanofiber mats on 3D printed scaffolds of rigid PLA did not result in sufficient adhesion between both materials, electrospinning PAN on soft TPU scaffolds led to the formation of a composite with good adhesion. In this way, the abrasion resistance of the nanofiber mats could be significantly enhanced. The hydrophilicity of the composite surface was slightly reduced, as compared to pure PAN nanofiber mats.

Since the good adhesion of PAN nanofibers on Filaflex TPU can be attributed to small residues of DMSO partially dissolving the scaffold surface, thus establishing a stable adhesive bonding, further research will investigate the influence of the distance between the high-voltage electrode wire and the substrate as well as the spinning voltage. Both of these parameters are considered to be significant influences on the amount of solvent residues at the time the nanofibers hit the substrate.

Comparing the results of this study with the previous one in which 3D printing was performed on nanofiber mats, the production order reported here has the advantage of being less technologically challenging and enabling combinations with flexible filament which could not be achieved in the previous study. On the other hand, the adhesion of PAN nanofibers electrospun onto a rigid PLA substrate was much too low for any technological application as a composite. Consequently, both production orders, starting either with 3D printing or with electrospinning, offer their own respective advantages and will be investigated in more detail in the upcoming studies, to obtain mechanically stable water filters containing electrospun nanofiber mats.
